# A Pilot Study of Opportunistic Chronic Kidney Disease Screening in Primary Care Using a Clinical Decision Support System

**DOI:** 10.3390/diagnostics16010008

**Published:** 2025-12-19

**Authors:** Maite López-Garrigós, Estanislao Puig, Selene Sánchez, Irene Gutiérrez, Maria Salinas, Alberto Ortiz, Emilio Flores

**Affiliations:** 1 Clinical Laboratory, Hospital Universitario de San Juan, 03550 San Juan de Alicante, Spain; lopez_marter@gva.es (M.L.-G.); gutierrez_ire@gva.es (I.G.); mariasalinaslacasta@gmail.com (M.S.); 2 Diagnostic Innovation in Laboratory Medicine Group, FISABIO, Avda. Cataluña, 21, 46020 Valencia, Spain; 3 Department of Biochemistry, Universidad Miguel Hernandez, 03202 Elche, Spain; 4 Centro de Investigación Biomédica en Red (CIBER) Epidemiología y Salud Pública (ESP), 28029 Madrid, Spain; 5 Primary Care, Department Alicante-Sant Joan, 03550 San Juan de Alicante, Spain; puig_est@gva.es (E.P.); sanchez_selgra@gva.es (S.S.); 6 Department of Nephrology and Hypertension, Universidad Autónoma de Madrid (IIS-FJD, UAM), 28040 Madrid, Spain; alberto.ortiz@uam.es; 7 Health Research Institute—Fundación Jiménez Díaz University Hospital, Universidad Autónoma de Madrid (IIS-FJD, UAM), 28040 Madrid, Spain; 8 RICORS2040-Renal, Instituto de Salud Carlos III, 28029 Madrid, Spain; 9 Departamento de Medicina, Facultad de Medicina, Universidad Autónoma de Madrid, 28049 Madrid, Spain; 10 Department of Clinical Medicine, Universidad Miguel Hernandez, 03202 Elche, Spain

**Keywords:** chronic kidney disease, primary care, clinical decision support system, opportunistic screening, early diagnosis

## Abstract

**Background/Objectives**: CKD affects over 10% of adults and is often silent, delaying diagnosis. Opportunistic primary care screening supported by clinical decision support systems (CDSSs) may improve detection with minimal burden. We evaluated the feasibility, diagnostic yield, clinical actions, and reagent costs of a CDSS-enabled, albuminuria-first program using eGFR. **Methods**: This one-year cross-sectional intervention screened all patients receiving routine laboratory tests at a primary care center using a CDSS integrating prior labs, medical records, and guideline rules. Eligibility required patients age 60–85 (Group 1) or 18–59 with hypertension, diabetes, or cardiovascular disease (Group 2). Eligible patients received urine albumin and eGFR testing with standard phlebotomy; abnormal findings triggered confirmatory tests. Outcomes were diagnostic yield, KDIGO risk stratification, referral patterns, and reagent costs. The CDSS surfaced prompts and pre-populated orders in the laboratory interface. **Results**: Of 7722 targets, 1892 (24.5%) were flagged (34.2% of Group 2, 7.9% of Group 1), and 1774 (93.8%) completed screening. We identified 104 new CKD cases (5.9%): 75% KDIGO moderate risk, 19% high, and 6% very high. Twenty patients (1.1%) met criteria for nephrology referral. Guideline-directed therapy was started or optimized in 90%, and 62.5% received a new CKD diagnosis code. Reagent costs averaged EUR 0.51 per person screened and EUR 11.14 per CKD case detected. Most cases were early-stage and manageable in primary care. **Conclusions**: CDSS-enabled opportunistic screening in primary care is feasible, acceptable, and low-cost. It identifies previously unrecognized CKD at modest expense, enabling early interventions that may slow progression and reduce cardiovascular events. Scaling with follow-up should assess long-term outcomes.

## 1. Introduction

Chronic kidney disease (CKD) is a major global health problem because of its high prevalence and its substantial contribution to morbidity, mortality, and healthcare expenditure [1,2]. Despite this impact, CKD is frequently asymptomatic and, therefore, remains undiagnosed until late stages, limiting the window for effective intervention [3]. Current KDIGO and national guidelines specify which individuals should be screened, yet implementation in everyday practice is uneven—particularly in primary care settings, where most patients at risk are first seen. Primary Care Centers (PCCs) are ideally placed to detect hidden CKD. Early identification can slow CKD progression, reduce cardiovascular events, and postpone renal replacement therapy [4,5,6,7].

Opportunistic screening—leveraging any blood or urine sample obtained for another purpose—can embed CKD detection seamlessly into routine workflows [8]. In Spain, the ENRICA study estimated a 15.1% prevalence of CKD across all stages, underlining the scale of undiagnosed disease [9]. National consensus documents now advocate a multidisciplinary PCC approach to CKD detection and early management [7,10]. In Spain, they recommend CKD screening from age 60 onwards as well as in the presence of high risk of CKD (hypertension, diabetes, or cardiovascular disease) [10]. Inclusion of age is likely linked to the long life expectancy in Spain, forecasted to become the longest in the world by 2040 [11], and differs from KDIGO, which does not consider older age a risk factor [4].

The clinical laboratory plays a fundamental role in the diagnosis of CKD, because KDIGO defines CKD by either persistently reduced estimated glomerular filtration rate (eGFR) or markers of renal damage, such as albuminuria present for ≥3 months [4,10]. Although albuminuria is a powerful prognostic marker and a mandatory criterion for full risk [12] stratification, it is still under-requested in Spanish primary care [13]. Semi-quantitative urine albumin strips are inexpensive, quick, and, when positive, can automatically reflexively trigger a quantitative urine albumin to creatinine ratio (UACR), yielding both clinical and economic advantages [14].

Artificial intelligence (AI) is transforming healthcare and clinical laboratories, and one of its applications is Clinical Decision Support Systems (CDSSs). These digital solutions are designed to assist healthcare professionals in making clinical decisions and can be instrumental in opportunistic screening to facilitate early CKD detection and management [15,16].

In a systematic review, Cheng Yeo et al. [17] reported that opportunistic CKD screening in adults is cost-effective: early detection in primary care improves outcomes and cuts costs by preventing progression to dialysis or transplant, directly supporting our proposed approach. However, there are different approaches to screening. Implementing an initial semi-quantitative approach that may automatically trigger quantitative albuminuria testing can lead to significant economic savings as compared to a quantitative-first approach by reducing the need for the more expensive and complex quantitative test.

We evaluated the feasibility and effectiveness of CDSS-enabled opportunistic CKD screening in primary care. Specifically, we implemented a novel CDSS algorithm in PCC patients using a semi-quantitative albuminuria-first approach and eGFR, and we measured its diagnostic yield, clinical actions, and reagent costs in a pilot study.

## 2. Materials and Methods

### 2.1. Study Design

We performed a one-year cross-sectional intervention (1 June 2023–30 June 2024) that enrolled all patients attending PCC who required laboratory testing for any clinical indication.

### 2.2. Setting

The pilot study was carried out at the San Juan PCC, serving a population of 26,129 inhabitants (52% female, and 19% aged over 65 years). The PCC employs 15 doctors and 24 nurses and handles 199,816 primary care consultations annually.

Our laboratory has implemented a CDSS as a rule engine (AlinIQ CDS v8.2; Abbott, Chicago, IL, USA) to manage pathways and actions. The CDSS allows identification of requests, including a specific test in current and recently made requests. In addition, the CDSS integrates with clinical databases, such as electronic medical records, to obtain information on all current and previous patient diagnoses. If certain conditions are detected in its rules engine, CDSS can generate alerts or add tests within a time frame of less than one second, allowing a real-time seamless integration into a busy PCC [16,18].

### 2.3. Study Patients

Table 1 outlines the inclusion and exclusion criteria for primary care patients within our population coverage area. These criteria pertain to individuals for whom a general practitioner (GP) has requested a laboratory check-up for any reason. The study was approved by the Hospital Ethics Research Committee (code 24/071).

### 2.4. Intervention

The intervention was designed and agreed upon with GPs and a nephrologist through two consecutive meetings. In the first meeting, a laboratory proposal based on guidelines [10] was analyzed, and in the second meeting, the initial results were presented to redesign the procedure and decide on key performance indicators (KPIs), based on patients’ outcome results. The intervention was sequentially implemented at a PCC, starting with a face-to-face meeting at the PCC, where a laboratory professional presented the project.

**Figure 1:**
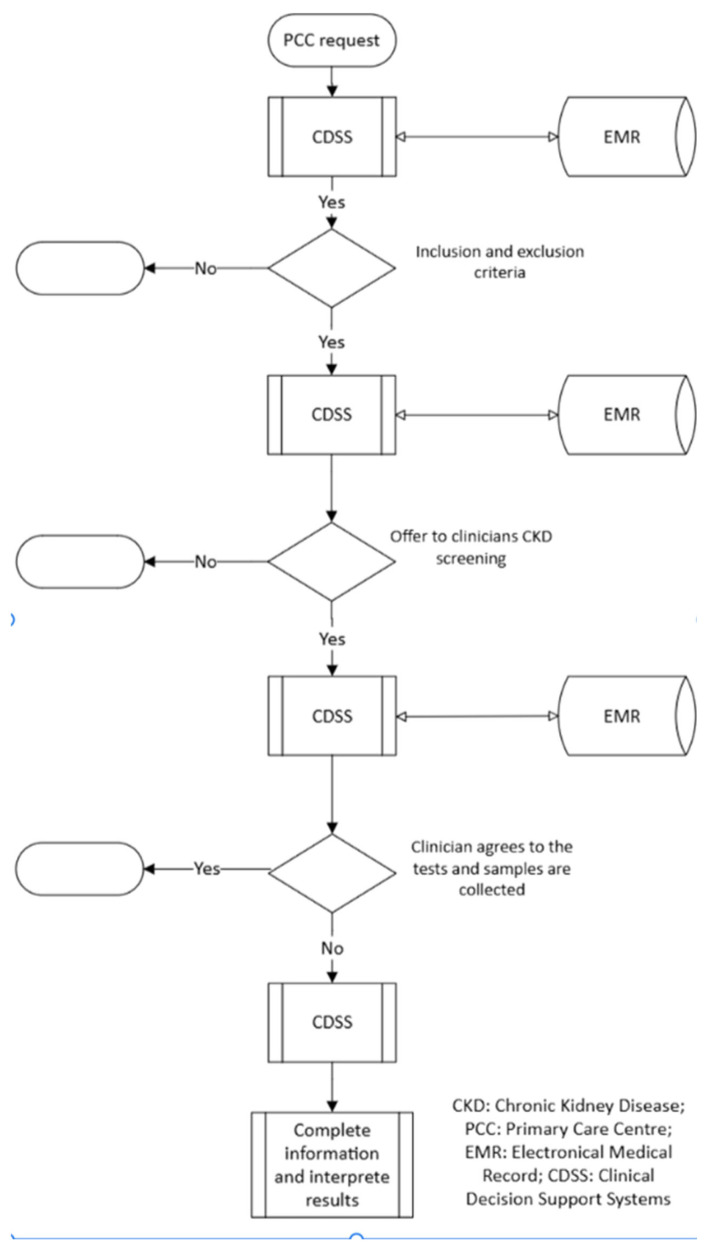
Workflow of opportunistic chronic kidney disease screening in primary care using a clinical decision support system. A real-time CDSS operationalized opportunistic CKD screening during laboratory ordering. For each request, it verified residency and risk-group eligibility, checked EMR diagnoses, and queried the LIS to avoid repeat screening within 12 months. When eligible, it prompted GPs via CPOE to offer screening and, upon acceptance, registered a UACR test. Using current guidelines, it computed KDIGO risk, flagged the need for confirmation, and recommended primary care versus nephrology follow-up. All outputs were appended as interpretive comments to laboratory reports.

Figure 1 illustrates the implemented workflow. The CDSS identified patients eligible for potential CKD screening based on inclusion criteria (Table 1) that implemented a Spanish National consensus document co-authored by 10 scientific societies, including nephrology, primary care, and laboratory medicine societies [10]. We assessed two target groups based on the inclusion criteria in Table 1. Group 1 included patients aged 60–85 years. Group 2 had patients aged 18–59 years with diabetes, hypertension, or cardiovascular diseases.

The CDSS executed the following real-time steps for every laboratory request:

1. Patient eligibility. Query of the population registry to confirm residence in the same health area of the study and membership of Group 1 or 2.
2. Clinical history check. Electronic medical record (EMR) search for existing diagnoses of CKD, diabetes, hypertension, or cardiovascular disease.
3. Previous screening. Laboratory Information System (LIS) query to determine whether CKD screening had been performed within the previous 12 months.
4. Test offer. Utilizing real-time connection to Computerized Physician Order Entry (CPOE), the CDSS offered GPs the option to screen for CKD. Upon agreement with the patient, the CDSS, through real-time connections to LIS and CPOE, facilitated the request of UACR testing, if not already requested.
5. Risk stratification. Using current guidelines [9], the CDSS calculated KDIGO risk, indicated whether confirmation testing was required, and recommended the appropriate level of follow-up (primary care vs. nephrology) for high-risk patients.
6. Reporting. All decisions and recommendations were documented in an interpretative comment appended to the laboratory report.

**Figure 2:**
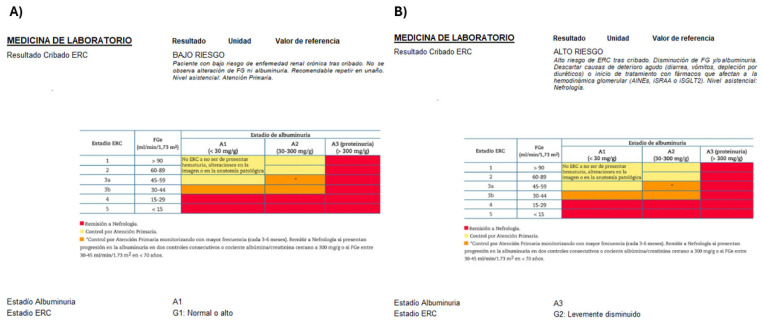
Laboratory report. Figure shows two examples of laboratory reports (example (**A**) is a low-risk report and example (**B**) is a high-risk report). It includes glomerular filtration rate categories and albuminuria categories. Additionally, a comment in the laboratory report recommended that patients at risk of CKD be followed up in primary care or nephrology, according to guidelines. The comments for (**A**): CKD screening result: LOW RISK. Patients at a low risk of chronic kidney disease after screening. No alteration in GFR or albuminuria is observed. It is recommended to repeat screening in one year. Level of care: Primary care. The comments for (**B**): CKD screening result: HIGH RISK. High risk of chronic kidney disease after screening. Decreased GFR and/or albuminuria. Rule out causes of acute deterioration (diarrhea, vomiting, and depletion due to diuretics) or the start of treatment with drugs that affect glomerular haemodynamics (NSAIDs, RAS inhibitors, or SGLT2 inhibitors). Level of care: Nephrology.

Figure 2 shows the laboratory report. It is based on KDIGO guidelines [4], as adapted by the Spanish consensus [10], and conveys the risk level with appropriate comments:

- “Low CKD Risk. A second screening is recommended in 1 year.”
- “Confirmation is needed through blood analysis in 1–2 weeks.”
- “Confirmation is needed through blood analysis in 1–3 months.”
- “Moderately increased CKD Risk.”
- “High CKD Risk.”
- “Very high CKD Risk.”

Moreover, the laboratory report (Figure 2) included glomerular filtration rate categories, estimated from serum creatinine, and albuminuria categories. Additionally, a comment in the laboratory report recommended that CKD Risk patients be followed up in primary care or nephrology, according to guidelines [9].

### 2.5. Laboratory Methods

Urine albumin and creatinine were first assessed semi-quantitatively on the automated UC-3500 analyzer (Sysmex, Kobe, Japan) using the Meditape UC-11A dipstick based on the protein error-of-pH indicator principle (tetrabromophenol blue) in combination with CMOS (Complementary Metal-Oxide Semiconductor) sensor-based reflectometry [14]. Previously established thresholds for urine albumin and creatinine were used to automatically trigger the quantification of urinary albumin and creatinine in the same sample and day. Quantitative urinary albumin was measured by immunoturbidimetry and creatinine by a kinetic colorimetric method on the Alinity C platform (Abbott Diagnostics, Chicago, IL, USA) [14].

### 2.6. Data Collection

We calculated the number of target patients. For each target subgroup, we identified the number of target patients, the number of patients offered CKD screening, the number who accepted, and the number in whom screening was completed. Newly detected potential CKD cases were stratified by KDIGO risk categories. Basic demographic characteristics were also collected.

CKD management actions were evaluated based on two outcomes: the percentage of patients that received a CKD-related treatment (angiotensin-converting enzyme inhibitors or angiotensin receptor blockers, sodium-glucose cotransporter 2 inhibitors) and the percentage of patients who included some new diagnostic-related with CKD (N18). We compared the number of new diagnoses with the same period of the previous year.

We also counted the number of UACR that were automatically requested, the number measured through strip analysis [10], and the number that needed quantification. The reagent costs for UACR were EUR 0.26 for strip analysis and EUR 0.62 for quantification. Costs were divided by the number of patients classified as KDIGO low and high-risk, and they were further divided by risk patients to evaluate two key performance indicators: cost per patient screened and cost per patient identified as high CKD risk.

### 2.7. Statistical Analysis

Demographic summary statistics were presented as quantity (N) and frequency (%) for categorical variables and mean ± SD or median (Q1–Q3) for quantitative data. Differences between groups were evaluated using the Chi-square test, with a two-sided *p* ≤ 0.05 considered significant. Statistical analyses were performed using Statistical Package for the Social Sciences (SPSS), Version 22 (IBM Corp., Chicago, IL, USA).

## 3. Results

### 3.1. Study Population and Screening Uptake

The target cohort comprised 7722 individuals, representing 29.6% of the registered inhabitants. Group 1 contained 4875 adults aged 60–85 years (63%), whereas Group 2 consisted of 2847 adults aged 18–59 years with ≥1 relevant comorbidity (37%).

The CDSS flagged 1892 (24.5%) patients for potential CKD screening based on the outlined criteria in one year. Among these, 1774 (93.8%) accepted the screening. The proportion of the target population selected for screening (Table 2) differed markedly between subgroups—34.2% in Group 1 versus 7.9% in Group 2 (*p* < 0.01). Acceptance rates among eligible patients also differed significantly (95.3% vs. 86.7%; *p* < 0.01). The principal reasons for noncompletion were patient or clinician deferral of screening (58%), failure to return a urine specimen (32%), and suspected urinary tract infection or hematuria (10%).

### 3.2. Identification of Patients with CKD

**Figure 3:**
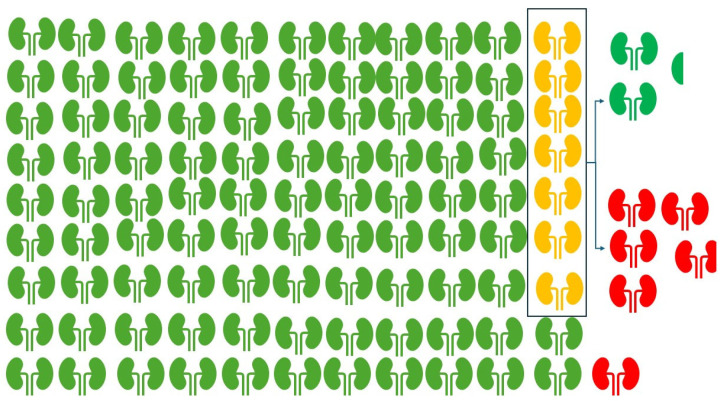
Classification of patients. Figure shows the classification of the patients before chronic kidney disease (CKD) screening. Green color: Patients were ruled out for CKD (94.1%); yellow color: required confirmatory testing (7.0%); and red color: Patient CKD diagnostic criteria (5.9%).

Among the screened cohort, 125 patients (7% of 1774) required confirmatory testing because guidelines require at least two abnormal results over three months. Follow-up testing showed that 45 (36.0%) did not meet the criteria for CKD, whereas 80 (64.0%) were classified as having CKD. Overall, among 1774 people screened, 1670 individuals (94.1%) were ruled out for CKD, and 104 (5.9%) met the CKD diagnostic criteria (Figure 3).

Sex and age differed significantly between the low-risk and risk groups (*p* < 0.01); low-risk patients were more often female (61% vs. 42%) and younger (mean 67.7 ± 9.0 years vs. 72.9 ± 8.0 years). According to clinical guidelines, 20 patients (1.1% of 1774, 19% of CKD cases diagnosed) were referred for nephrology follow-up, while 84 patients (4.7% of 1774, 81% of CKD cases diagnosed) were managed in primary care.

Table 3 summarizes risk stratification among the 104 patients with confirmed CKD. Seventy-eight individuals (75.0%) were categorized as KDIGO “moderately increased” risk, 20 (19.2%) as “high” risk, and six (5.8%) as “very high” risk. Most (n = 100) cases arose in the age-based Group 1, while only 4 of 226 (1.8%) participants in Group 2 (risk factors other than age) had CKD, all with a moderately increased risk. Mean estimated eGFR and UACR deteriorated stepwise across risk categories, and the proportion of patients with pathological eGFR or albuminuria rose correspondingly.

### 3.3. Clinical Actions Resulting from Screening

In the 104 patients identified as having CKD, clinical actions were evaluated. A total of 90 (90.4%) patients received a new CKD-related treatment. Only 65 (62.5%) patients had a new diagnosis of CKD recorded in EHR; however, this alone was already 2.6-fold higher than in the same period of the previous year (n = 25).

### 3.4. Cost Analysis

The cost analysis for CKD screening, based on reagent costs, showed an average expense of EUR 0.51 per screened patient. Over the study period, the total expenditure on CKD screening reagents was EUR 904.74 for the 1774 screened patients. Cost per identified CKD patient (CKD risk) was EUR 11.14.

## 4. Discussion

The findings from the study demonstrate the feasibility and effectiveness of opportunistic CKD screening in primary care settings. The high acceptance rate indicates that the strategy was well received by patients and GPs. A significant proportion of screened patients were identified as having high CKD risk, highlighting the prevalence of undiagnosed CKD in the primary care population. The structured screening protocol facilitated the timely identification and management of patients at risk for CKD, allowing for early intervention and potential reduction in CKD progression and complications.

Integrating CDSS with EMR and CPOE facilitates seamless CKD risk assessment and management, highlighting its role in improving patient care [3]. This integration of CDSS with CPOE and EMR systems in real-time is crucial for the success of CKD screening programs. The CDSS helps in identifying at-risk patients quickly and accurately, ensuring that appropriate tests are ordered and evaluated according to established guidelines [4,7]. The laboratory, by means of digital solutions, plays a pivotal role in diagnosing CKD, providing critical data that informs patient risk stratification and management plans. By applying guidelines in real-time, the CDSS ensures that interventions are both timely and appropriate, leading to better patient outcomes [7,8,18,19].

Within a single year, the CDSS identified nearly 25% of the target population who required screening that had not been requested in primary care (PCC). However, in subgroup analyses, younger individuals with comorbidities—predominantly men, with a mean age of 51 years—had markedly lower access to screening (<8%). This likely reflects the lower frequency with which younger adults attend primary care compared with older adults, which is consistent with previous studies, such as the KEEP program [20]. Overall, the results support the age-based approach proposed by the Spanish consensus and recently supported by other scientific societies and organizations [11,21].

The CDSS identified previously unrecognized CKD in 104 of 1774 screened patients (5.9%). This yield is lower than that reported in other opportunistic screening studies, likely for two reasons: our selection criteria strictly followed the Spanish primary care guidelines, and the system was triggered only in patients who were already being visited in primary care, limiting the general population. The risk profile of the detected cases is similar to prior cohorts, with a higher proportion of men and older age. Notably, many of the new diagnoses showed noncritical eGFR declines (predominantly KDIGO G2–G3a), underscoring early-stage detection. Albuminuria (assessed by UACR) was the earliest detectable abnormality in our cohort, aligning with KDIGO recommendations that emphasize albuminuria for risk stratification and therapeutic decision-making [22]. An albuminuria-based strategy has been supported by the European Renal Association ABCDE framework for assessment of cardiovascular–kidney metabolic health, where A stands for albuminuria, B for blood pressure, C for cholesterol, D for diabetes (glycemia), and E for eGFR [23,24], based on the 2021 European Society of Cardiology guidelines for the prevention of cardiovascular disease [25]. The optimal integration of albuminuria screening into clinical practice is a focus of intensive research [26,27,28].

Regarding decision-making, in our cohort, 90.4% of newly identified CKD cases received a guideline-directed intervention, most within the primary care setting. Evidence from clinical trials suggests the initiation of treatment triggered by an albuminuria-based diagnosis of CKD when GFR is still preserved may delay the need for kidney replacement therapy by up to 27 years [29]. The Danish ATLAS primary care study reported that 62% of patients received guideline-directed interventions [30]. By contrast, only 62.5% of our patients were assigned a new CKD diagnostic code, which falls below the median 73.9% coding rate seen across the UK’s general practices in a large population study where higher coding was linked to better outcomes [31].

Our cost analysis shows that opportunistic CKD screening is cost-effective, with low costs per patient screened and per risk case identified; notably, most UACR tests did not require quantitative confirmation, markedly improving efficiency. These results close the loop of our earlier work: in 2012, we documented an under-requesting of UACR [13]; in 2018, we demonstrated that semi-quantitative strip testing reduces laboratory costs [15]; and here, we show that reinvesting those savings enables the affordable detection of previously hidden CKD in primary care. This stepwise strategy improves CKD management while generating meaningful health system savings, which is in line with prior evidence supporting the economic and clinical benefits of early detection [17]. Implementing systematic, opportunistic screening in primary care can, therefore, enhance outcomes and optimize resource utilization.

Opportunistic CKD screening in primary care—supported by a CDSS integrated with EMR/CPOE—enables earlier detection, timelier intervention, and lower downstream costs, benefiting patients, clinicians, and the health system [1,4,5,32]. Economic evaluations (e.g., Cheng Yeo et al.) indicate that early CKD detection is cost-effective in the general adult population, while van Mil et al. highlight the remaining evidence gaps but broadly support this direction; home-based albuminuria screening can complement opportunistic primary care strategies [28].

## 5. Conclusions

In conclusion, high acceptance and substantial high-risk yield support embeds real-time, CDSS-enabled opportunistic CKD screening into routine primary care. This cost-effective strategy must be underpinned by robust urinalysis technology, as it improves early intervention, aids evidence-based management by GPs, and may lessen the burden on the health system. Furthermore, it allows initiating early therapy for CKD at the primary care level without overloading the healthcare system, as these patients were already being followed by primary care. Future work should scale the model to other PCCs, quantify long-term effects on CKD progression, consider costs and resource use, and address the persistent challenge of consistent early-stage screening.

## Figures and Tables

**Table 1:** Inclusion and exclusion criteria.

Inclusion Criteria	Exclusion Criteria
GP requested a lab test for any reason. GP agrees with the screening. Primary care patients of our population area (Health Department 17). Age-based subgroup (Group 1): All patients aged ≥ 60–85 years. Comorbidity-based subgroup (Group 2): Patients aged ≥ 18–59 years having specific comorbidities by ICD-10: Patients: ○ I10 Essential (primary) hypertension. ○ Diabetes: ▪ E10 Type 1 diabetes mellitus. ▪ E11 Type 2 diabetes mellitus. ▪ E13 Other specified diabetes mellitus. ▪ E14 Unspecified diabetes mellitus ○ Cardiovascular disease: ▪ I20 Angina pectoris. ▪ I21 Acute myocardial infarction. ▪ I22 Subsequent acute myocardial infarction with ST elevation (STEMI) and without ST elevation (NSTEMI). ▪ I23 Ongoing complications, following ST-elevation myocardial infarction (STEMI) and non-ST-elevation myocardial infarction (NSTEMI) (within 28-day period). ▪ I24 Other acute ischemic cardiac diseases.	GP disagrees with the screening. Known diagnosis of chronic kidney disease (ICD 10 code N18). Previous CKD screening performed within the past 12 months. Pregnancy.

GP: General practitioners; CKD: Chronic kidney disease; ICD-10: International Classification of Diseases 10.

**Table 2:** Demographic data of the flagged and screened population.

	Flagged Population			Screened Population		
Group	Group 1	Group 2	All	Group 1	Group 2	All
Patients	1666	226	1892	1578	196	1774
Male sex (%)	43%	54%	44%	43%	53%	44%
Age (years)	70.3 ± 6.7	50.4 ± 8.1	67.9 ± 9.5	70.4 ± 6.7	51.0 ± 7.4	68.3 ± 9.1

Group 1: Age-based subgroup: all patients, age ≥ 60–85 years. Group 2: Comorbidity-based subgroup: Patients having specific comorbidities according to the International Classification of Diseases 10 (hypertension, diabetes, and cardiovascular disease): patients aged ≥ 18–59 years.

**Table 3:** Laboratory data of CKD cases stratified by the KDIGO heatmap.

	Moderately Increased Risk	High Risk	Very High Risk
Patients (n)	78	20	6
Male sex (%)	65%	62%	50%
Age (years)	72.5 ± 8.3	74.8 ± 6.7	71.8 ± 9
eGFR (mL/min/1.73 m^2^)	70.3 ± 16.3	66.3 ± 06.3	41.0 ± 8.7
UACR (mg/g)	66.3 (IQR 30.9–140)	392.1 (IQR 313.4–528.5)	868.6 (IQR 42.5–1282.5)
eGFR G3–G5, n (%)	31 (39.7%)	7 (35.0%)	6 (100%)
UACR A2–A3 (>30 mg/g), n (%)	47 (60.3%)	16 (80.0%)	5 (83.3%)

eGFR: glomerular filtration rate; UACR: urine albumin to creatinine ratio. IQR: interquartile range (distance between the 25th and 75th percentiles).

## Data Availability

The dataset is available on request from the authors. The raw data supporting the conclusions of this article will be made available by the authors upon reasonable request.

## Abbreviations

The following abbreviations are used in this manuscript:

CKD	Chronic kidney disease
PCC	Primary care center
eGFR	Estimated glomerular filtration rate
UACR	Urine albumin to creatinine ratio
CDSSs	Clinical decision support systems
KPI	Key performance indicator
EMR	Electronical medical record
LIS	Laboratory Information System
CPOE	Computerized Physician Order Entry
